# Estimating Haplotype Frequency and Coverage of Databases

**DOI:** 10.1371/journal.pone.0003988

**Published:** 2008-12-22

**Authors:** Thore Egeland, Antonio Salas

**Affiliations:** 1 University of Oslo, Institute of Forensic Medicine, Oslo, Norway; 2 Oslo University College, Oslo, Norway; 3 Unidade de Xenética, Instituto de Medicina Legal, Facultad de Medicina, Universidad de Santiago de Compostela, Galicia, Spain; University of Utah, United States of America

## Abstract

A variety of forensic, population, and disease studies are based on haploid DNA (e.g. mitochondrial DNA or Y-chromosome data). For any set of genetic markers databases of conventional size will normally contain only a fraction of all haplotypes. For several applications, reliable estimates of haplotype frequencies, the total number of haplotypes and *coverage* of the database (the probability that the next random haplotype is contained in the database) will be useful. We propose different approaches to the problem based on classical methods as well as new applications of Principal Component Analysis (PCA). We also discuss previous proposals based on saturation curves. Several conclusions can be inferred from simulated and real data. First, classical estimates of the fraction of unseen haplotypes can be seriously biased. Second, there is no obvious way to decide on required sample size based on traditional approaches. Methods based on testing of hypotheses or length of confidence intervals may appear artificial since no single test or parameter stands out as particularly relevant. Rather the coverage may be more relevant since it indicates the percentage of different haplotypes that are contained in a database; if the coverage is low, there is a considerable chance that the next haplotype to be observed does not appear in the database and this indicates that the database needs to be expanded. Finally, freeware and example data sets accompany the methods discussed in this paper: http://folk.uio.no/thoree/nhap/.

## Introduction

Haploid genetic data are commonly used in population genetics (e.g. [1], [2]), forensics (e.g. [3], [4]), and genetic studies of disease [5]. The most popular haplotypic data in the literature are from the non-recombining part of the Y-chromosome and the mitochondrial DNA (mtDNA). These markers are transmitted from parents to the offspring in a patrilineal (Y-chromosome) or matrilineal (mtDNA; see [6] for a recent discussion) way as pure haplotypic blocks. In a forensic context, these markers are routinely used in many applications. For instance, when biological material is degraded and only mtDNA may be analyzed, or in a rape case where the Y-chromosome is often more helpful than other markers; such data may serve to strengthen the case against the suspect or the suspect may be cleared. Other forensic applications involve identification ranging from maternity/paternity cases to larger pedigrees, perhaps extending over several generations. Such applications may extend well beyond the forensic field. For example, mtDNA and the Y chromosome have played a central role in disentangling the ancient and recent past of human populations and their demographic movements. In addition, mtDNA has also been related to a plethora of complex diseases.

The methods proposed here can also be applied to markers of a different nature. For instance, there are various methods available for reconstructing haplotypes from unphased genotype data (e.g. [7] or HapMap: http://www.hapmap.org). There is vast number of other references that could be mentioned; the HLA-complex is an important example of autosomal data covering all of the mentioned areas.

The aims of the present article were motivated by the mentioned applications. For example, it is frequently desirable in population or forensic genetics to estimate the fraction of unseen haplotypes in a population given a sample of a particular size. Similar problems have been studied extensively with diverse applications in mind. An early paper discussing statistical approaches for estimating the number of species in a population is [8]. A general review is given by [9] whereas more specialized reviews are provided by [10] (for the case when the population size is known) and [11] (based on empirical Bayes methods of [12]). Huang and Weir [13] discuss the estimation of the number of alleles using coverage methods [14].

The main problem described loosely above may now be formulated precisely as follows: There is an extremely large number of haplotypes (*n*) in a population. For modeling purposes we will assume this number to be infinite. There are, however, some papers dealing with the finite case (e.g., [10]) but this less common approach will not be followed here. The number of different haplotypes (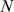) is smaller, but typically large in the applications we have in mind. A sample of haplotypes is available from this population. Based on this sample and possible further extended samples we would like to address the following questions:

1. What is the total number of different haplotypes (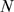) in the population?
2. What is the coverage (*C*)? Specifically, if we unrealistically assume 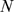 to be known and equal to 1000 and our sample contains 600 different haplotypes, the coverage will be 0.6.
3. What is frequency of a previously unseen haplotype? While this by necessity is a difficult and speculative question, several applications, including forensic, require a specific estimate.

Comparing this paper to the existing literature, we first note that haploid data has not been considered extensively previously, and the treatment of this data is particularly complex. An understanding of phylogenetic/phylogeographic features of haplotype data is required and should be accounted for. Regarding problem 3 above we implement a Principal Component procedure and argue that substantial features of the data are incorporated through this model. More generally, there is an extensive literature dealing with problems 1, 2 and 3 above, and our ambition has been to focus on approaches particularly suitable for haplotype applications.

Answers to these problems have direct implications for sample size problems and design issues more generally and also estimation of haplotype frequencies. To our knowledge, a pragmatic solution to this problem, when applied to haploid data, is still not available. We therefore provide relevant examples based on simulations as well as mtDNA and Y-chromosome data and also freely available software running in R (http://www.r-project.org/).

## Analysis

### Review of methods related to the coverage of a given database

There is a large literature of statistical papers dealing with problems resembling those we are addressing. The generic problem addressed is often referred to as ‘Species richness estimation’ and classical references include [8], [15] while more recent reviews are provided in [9], [16]. In this section we provide the required notation and also review the models we consider to be most suitable for the applications we have in mind.

Let *n* denote the total number of haplotypes in the database, and *D* the number of different, unique haplotypes. 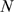 is the total, unknown number of unique haplotypes. Let *p_i_* be the probability that a haplotype belongs to the ith class and *X_i_* the number of elements of the ith class in the sample, i = *1*,*…*,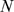. Furthermore, *f_j_* is the number of haplotypes observed exactly *j* times (which corresponds to the frequency spectrum of haplotypes in population genetics). The sample coverage, *C*, is defined as the sum of the probabilities of the observed classes, i.e.,
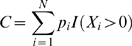
(1)where *I* denotes the indicator function. Observe that when all haplotypes are sampled, *C* equals the sum of all frequencies and so *C = 1*.

Assuming unrealistically that all haplotypes are equally frequent, a first estimate of the total number of unique haplotypes 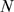 is
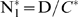
(2)where the coverage defined in (1) is estimated as
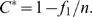
(3)


The latter estimate is also valid without the assumption of equal frequency but *f_1_* should be large for it to be accurate; the accuracy increases with increasing *f_1_* according to Good [15], who first presented the formula giving credit to the famous mathematician and computer scientist Alan Turing. Moreover, the probability that the next haplotype is new in the sense that it is not in the database is *1-C = f_1_/n*. If all haplotypes of the database appear exactly once, this probability is 1 since then *f_1_ = n*. It is intuitively reasonable that this probability should be close to 1 in this case, but perhaps not identical to 1, and [15] presents improved results that account for this. However, for our purposes these improved results are of little relevance and formula in (3) appears to solve the problem well. A similar comment applies to the opposite extreme occurring when *f_1_ = 0*.

A corollary to Theorem 1 of [17] gives the following asymptotic 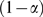 confidence interval for *C*

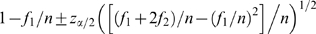
(4)where 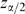 is the usual percentile based on the normal distribution. Below we use 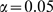 and 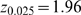.

We next present extensions based on Proposition 1 in [14]. The initial estimate (2) is increased by a term depending on the distribution of the class frequencies as measured by 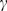, the squared coefficient of variation. In [14] it is shown that reasonable estimators are of the form
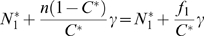
(5)and the authors propose estimators, denoted 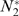 and 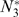, based on two different estimators of 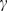 defined in Equations (2.12) and (2.13) of [14] respectively and reproduced below
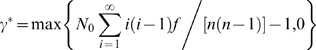
(6)

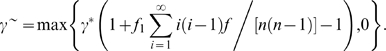
(7)


The estimator (2) may work well in cases where there are few prevalent haplotypes while the majority of them are rare. A standard trick described in [18] improves on (2) and also the two other estimators. The idea is to separate the observed haplotypes into two groups: *abundant* and *rare*. Abundant haplotypes are those that are observed more than *k* times and that are likely to appear in any reasonable sample. There appears to be a consensus on setting *k = 10* [13], [18]. The rare haplotypes are those that occur less often. With obvious notation, we may then write *D = D_abun_+D_rare_*. Note that 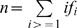. Following this approach *n* in (3) is replaced by 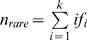 and the sums in Equations (6) and (7) are stopped at *k*. The estimator (2) then becomes
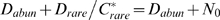
(8)implying modifications for the two other estimators as well. The practical importance of this modification is that the estimators become more stable and robust.

#### Example 1

The data for this example are shown in Table S1 (supplementary data). There are four binary sites and 30 haplotypes. The first haplotype, haplo1, is a singleton, the second is the most prevalent occurring 12 times while there are four copies of the third (haplotypes 3,4,16 and 18). We will use this data to exemplify the previous formulae and also our freeware implementation http://folk.uio.no/thoree/nhap/. There is too little data for this example to have any practical interest and in addition, the asymptotics are not likely to work. If examples of this magnitude are of practical interest, approaches following [10] may be better suited. The basic input data should be a matrix where lines correspond to haplotypes and columns to sites. The data in this example is in binary format but the sites could also be arbitrary integers, as is the case for the Y-chromosome data of supplementary Table S2. There are also other parameters, but the default values can and will be used for this example. This in particular implies that *k = 10*, i.e., haplotypes occurring less than 10 times (haplo2 in our data) are considered rare. There are 30 haplotypes, nine of which are unique. These nine unique haplotypes consist of *D_rare_* = 8 rare haplotypes and *D_abund_ = 1*. There are 12 copies of the abundant haplotypes (the one occurring 12 times). Furthermore
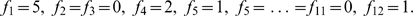



Consider first the coverage estimate in (3), i.e., 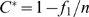. If there is no cut-off, then *n* = 30. In our case the cut-off is 10 and the sample size corresponding to those haplotypes occurring 10 times at the most is 30−12 = 18. The coverage estimate therefore becomes 1−(5/18) = 0.72. The 95% confidence interval can be calculated from (4) as
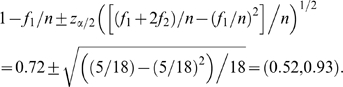



The first abundance estimator, 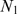 is given by (8) when there is a cut-off, i.e.,
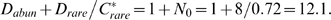



It remains only to demonstrate the last two abundance estimates. For this purpose, we first find 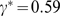 using (6). From this,
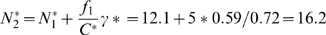



Finally, 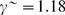 based on (7) and
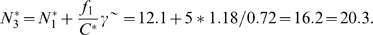



In this example there are at most 2^4^ = 16 different haplotypes and we see that 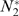 is close to this value. However, as mentioned previously, this example is too small to serve anything but illustration purposes.

#### Example 2

The next example uses simulated data based on *the coalescent*, see [19] for a review. 5000 mtDNA profiles were simulated under varying assumptions using Marchini's R-package *popgen* (http://www.r-project.org/). The mutation rate (θ = 2*Eμ*; where *μ* is the mutation rate per gene per generation and E is the effective population size) was varied in the *treesim* function. We consider these 5000 profiles to be complete data, i.e., all individuals have been typed. Then, for various sample sizes (50, 100, 400 and 1600) the number of unique haplotypes was estimated using the estimators 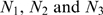, and the resulting estimators were compared to the true value based on the 5000 profiles. The result of a single arbitrarily selected simulation is provided in Table 1. For instance, the last line of this table shows that the sample of 5000 contains 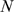 *= 412* different haplotypes. The coverage is estimated at 0.87. The 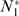, 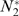 and 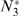 estimates are 349, 410 and 444 respectively. This reveals a general and expected feature summarized by 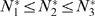, but these inequalities do not hold generally. For small sample sizes, all estimators underestimate and 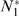 almost always underestimates. Table 2 compares accuracy using the root mean squared error based on 100 simulations.

**Table 1:** Performance of different approaches to the estimation of the number of different haplotypes based on simulated data.

θ	N	Sample	N^*^_1_	N^*^_2_	N^*^_3_	D	C^*^
10	66	50	23	26	28	19	0.84
10	66	100	33	45	56	28	0.84
10	66	400	50	57	62	45	0.88
10	66	1600	67	74	79	61	0.84
50	222	50	64	91	124	33	0.52
50	222	100	72	90	103	52	0.72
50	222	400	136	162	178	115	0.83
50	222	1600	191	215	227	175	0.89
100	412	50	215	506	1173	43	0.20
100	412	100	148	172	194	71	0.48
100	412	400	234	293	339	179	0.76
100	412	1600	349	410	444	311	0.87

A total of *n* = 5000 profiles were sampled from the coalescent for varying *θ ( = 2Eμ* where *μ* is the mutation rate per gene per generation and *E* the effective population size). The column ‘N’ gives the number of different haplotypes in this sample, the quantity to be estimated. The column ‘Sample’ shows the sample sizes used. Next follows the estimators 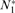, 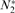 and 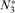. *D* gives the number of different observed haplotypes and is followed by the coverage estimate.

**Table 2:** Accuracy of estimators of the number of different haplotypes measured by the root of the mean squared error assessed by 100 simulations.

Sample	N^*^_1_	N^*^_2_	N^*^_3_
50	39.5	34.5	34.2
100	34	28.3	26.1
400	21.8	17.3	16.2
1600	8.7	6.4	10.3
50	159.65	143.76	131.87
100	148.38	123.47	105.3
400	100.58	71.44	54.09
1600	38.33	17.29	18.84
50	269.71	250.71	265.69
100	252.11	215.64	186.23
400	188.13	128.37	86.92
1600	73.58	23.75	33.31

Each simulation was carried out as in Table 1.

Table S2 presents an example based on Y-chromosome data that shows that large data sets can be easily handled and sites need not be binary.

#### Example 3

This example considers the ten databases of Table 3: Germany (*n* = 1314; [20], [21]), Iceland (*n* = 396; [22]), Mozambique (*n* = 306; [1]), Portugal (*n* = 540; [23], [24]), Basque (*n* = 171; [25], [26], [27]), Catalonia (*n* = 118; [28]), and Galicia (*n* = 135; [24], [29]). For instance, there are 1314 German samples and the coverage is estimated as explained above at 0.59. On the other hand, there are 396 Icelandic samples and the corresponding coverage is 0.77. So the Icelandic sample provides better coverage than the German sample despite being smaller (but not smaller compared to population size and perhaps population heterogeneity).

**Table 3:** Haplotype estimates from several population datasets.

pop	n	D	n.single	C	N^*^_1_	N^*^_2_	N^*^_3_
Andalucia	50	39	33	0.34	115	254	541
Basques	171	68	46	0.59	114	195	313
Catalonia	118	79	67	0.32	248	398	620
Galicia	135	76	58	0.43	177	217	256
Germany	1314	462	309	0.59	772	1333	2142
Icelandic	396	111	59	0.77	142	210	283
Mozambique	306	111	72	0.63	174	295	462
PortCent	160	93	74	0.42	219	378	621
PortNorth	184	106	79	0.45	234	288	342
PortSouth	196	113	88	0.43	260	392	564
Spain	474	203	147	0.55	365	695	1241
Portugal	540	242	162	0.58	411	632	903
Iberia	1014	383	261	0.58	650	1018	1488

The first sample (Andalucia) consists of 50 mtDNA HVS-I profiles, of which 39 are different. There are 33 singletons and so the fraction of unseen haplotypes is estimated as 33/50 = 0.66 and the coverage is 0.34. 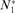, 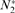 and 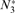 are different estimates of the number of haplotypes as explained in the text. The last three lines lump data from previous lines.

A useful result [11] is available along different lines. Assume we extend the sample by a fraction *t*. Then the expected number of new haplotypes is
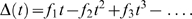
where, as previously, *f_j_* is the number of haplotypes seen precisely *j* times. The formula is valid for *t≤1* and *t = 1* corresponds to a doubling. The above formula is studied in greater detail in [30].

#### Example 4

Consider Table 3. There are 540 Portuguese samples of which 242 are unique. Doubling the database to 1080 corresponds to inserting *t = 1* into the above formula. The expected number of new haplotypes becomes
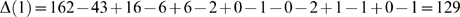
and so based on a sample of 1080 we would expect to see 242+129 = 371 different haplotypes. The above calculation excludes the most prevalent haplotype, the one occurring 113 times.

### Rare or unseen haplotypes

#### Estimation of unseen haplotype frequencies: general considerations

We would like to make the database sufficiently large to include all haplotypes having frequency *q* or higher. The simplest mathematical solution can be provided in case no data have been collected and there is no prior information we can use. Although this is not the case (since data are actually available), Plaza et al. [31] went ahead with a simplistic solution in a case of a mtDNA study carried out in an Angolan sample; briefly, the authors aimed to provide an upper bound of Khoisan (historical) demographic influence in their typically bantu Angolan populations given the fact that no Khoisan specific lineages have been detected in their sample: “*the probability of not finding a particular lineage that is present in a population at frequency of f in a sample of size n is given by α = (1−f)^n^*”. For a given *f*, *n* can be determined to ensure that *α* exceeds a specified lower limit, say 0.95. According to [31]“*…the maximum contribution of Khoisan lineages to Angolans is compatible with the observation that the absence of L0d and L0k in a sample of 44 Angolans would be less than 10.8% (for α = 0.95)*”. This estimate is however too simplistic. For instance, any unseen lineage takes the same value, so we would obtain the same probability estimate for a Khoisan lineage as for e.g. an East Asian or European one in the Angolan population. This estimate is also strongly dependent on sample size. In general, there is no way to know how close this value is to the true one, but upper limits can still be useful.

#### Classical approaches to the estimation of unseen haplotype frequencies

If a haplotype is never seen in the database, the classical frequentistic estimate will be 0 and will typically lead to unreasonable or impossible statistical analysis. For instance, in forensics, likelihood ratios may become infinite. For association studies, odds ratios may similarly be impossible to calculate. Different suggestions have been proposed in the literature to avoid such unfortunate consequences. If all haplotypes appear in the database, there are no unseen haplotypes and there is no problem. In practical cases, coverage is not complete and the ultimate goal is to provide an estimate as close as possible to the one we would observe in the hypothetical situation of complete coverage. A practical proposal is to let
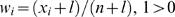
(8)where the haplotype is observed x_i_ times in a database of size *n*. The choice *l* = 1 amounts to adding the unseen haplotype to the database while *l* = 2 corresponds to adding both the defendant and culprit profile. This topic is discussed in Section 6.3.1 of [32] and in [33]. At any rate, the specific choice for *l* should be of minor importance. The frequencies must add up to unity as ensured by (8).

It is also possible to estimate the frequency of an unseen haplotype (or any haplotype for that matter) by simulating from the coalescent. The examples performed [34] indicate that the estimator (8) works well with *l = 1*. However, (8) tends to overestimate the true value for rare haplotypes by a relatively large amount since (8) corresponds to the case where the unseen haplotype appears as the next sample.

The problem of unseen haplotypes can be approached based on the above methods in a way which allows phylogeographic knowledge to be incorporated and this is discussed in the next section. Here, we mention briefly a simplistic approach: Assume the coverage is *C* and that there are *u* unknown haplotypes with unknown frequencies *p_1_*,*…*,*p_u_*. If these unknown frequencies are all the same, then their common estimate is 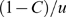. It may appear unreasonable to assign higher frequency to an unseen haplotype than one seen once and some modification may be required and 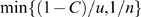 seems reasonable.

#### Principal Component Analysis applied to the estimation of haplotype frequencies

Estimation of haplotype frequencies depends heavily on the sample size, and ignoring phylogenetic or phylogeographic circumstances could lead to overestimation. For example, a typical sub-Saharan haplotype is probably rare in Andalucia (South Spain) in comparison with many other haplotypes that are probably relatively common in this region but that still remain unsampled due to the stochastic nature of the sampling process. Thus, based on the previous classical approach, we estimate the fraction of unseen haplotypes in a sample of 50 Andalucians as 0.66 (Table 3), but note that any unseen sample, either belonging to some typical European haplogroup or sub-Saharan one, will receive the same frequency, namely approximately 1%. For a European haplotype, this frequency could be realistic, but probably not for a typical Asian or sub-Saharan one.

In a typical forensic genetic context, a common scenario could be that of an immigrant suspect carrying a very rare haplotype with respect to the reference population where the crime was committed. The use of the local database, in which the haplotype carried by the suspect is very uncommon, will probably overestimate its frequency. It could be argued that the use of local databases is always conservative in this context, and this would benefit the suspect (benefiting therefore his/her innocent presumption). However, such a conservative interpretation could also be unfair if the suspect is responsible for the crime. ‘Extreme’ interpretations of the DNA test in forensic genetics are always undesirable, and here we just propose an alternative way that could contribute (among other medical and population genetics applications) to improve the evaluation of the DNA test, but not without warning potential users about the fact that there is probably not a single universal solution for routine casework, and that every single case will probably require particular treatment. Therefore, these estimates have to be taken with caution. Two intuitive considerations could help to interpret these estimates: i) a certain threshold for the fraction of unseen haplotypes (e.g. 10%) could be required beforehand in order to consider that the frequency estimate of a given haplotype is reliable, and ii) phylogenetic and phylogeographic information could be useful in order to evaluate how far the unseen haplotype is (in some sense) from the database. It would in fact be desirable to ‘weight’ the haplotype frequency estimate by incorporating phylogenetic considerations into a mathematical model, but the most intuitive way, namely, the use of genetic distances, is not straightforward for e.g. mtDNA data. In [35] a distance based on the minimal number of mutational steps was proposed for Y-chromosome data. We here propose a method based on Principal Component Analysis (PCA).

Assume the frequency of an unseen haplotype is
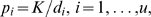
(9)where *K* is a constant determined by the requirement that these frequencies should add up to *1-C*, *d_i_* is the distance, in some sense, from the haplotype to the ‘population’ and *u* is the number of unseen haplotypes. Some assumptions and modelling are required to calculate these distances and we will use PCA. We explained and exemplified PCA on similar data for different applications in [36] and we refer to that paper for background and more general references. We will use ordinary Euclidean distance in the PCA space, i.e.,
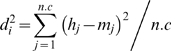
(10)where *n.c* is the number of principal components; *h_j_* and *m_j_* are the *j*-th coordinate of the haplotype and the mean in PCA-space respectively. For the simple case when there are two principal components (*n.c = 2*) there is a simple geometrical interpretation corresponding to the usual distance measure. Our practical PCA implementation uses the *prcomp* function of R.

If the distances based on all unseen haplotypes are available (which is unlikely), *K* can be calculated as
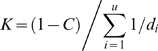
and the required frequency is readily available from (9). The problem is also solved if 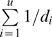 and thus *K* can be estimated by other means. Unfortunately, such solutions will normally not be available and in the example below we instead choose a pragmatic solution. Our basic assumption is that the naïve upper bound should not be exceeded. If the distance from the haplotype for which a frequency estimate is required is below the average of the internal PCA distances, *d̅*, we use this upper bound, i.e., *1/(1+n)*, and otherwise we use the following version of (9):
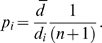
(11)


The above equation has an intuitively appealing interpretation as the traditional estimate is corrected by a factor accounting for phylogeographical features through PCA-distances.

#### Example 5

The first part of this example is based on simulated data. The data is simulated to share some features with the real data of the latter part of the example. Two populations are simulated, each with *n* (50,100 and 400) individuals. There are 100 sites that may vary, so a haplotype is a binary vector of length 100, e.g., (0,1,0,…,1). There are various ways such data can be simulated. In Example 2 we used the coalescent. Here a simpler approach is more convenient: 0 and 1 are simply simulated independently with probabilities *1-q* and *q*. In this way it is easier to describe and characterise the data. We consider here three scenarios. For the first scenario there is a relatively small difference between the populations (*q* = 0.1 and 0.3 respectively) whereas the difference between the populations is larger for scenario 2 (*q* = 0.1 and 0.5) and scenario 3 (*q* = 0.1 and 0.9). The results of the simulations are summarised in Table 4. The naïve bound provides a comparison with what we consider to be the most usual alternative to the approach we have described, namely the estimate *1/(n+1)*. Next there is a need for unseen haplotypes, test data. For the columns ‘fraction1’ and ‘median1’ we have considered haplotypes in population 2 which are unseen in population 1. The estimate of the unseen frequency is calculated according to (11). The frequency of these estimates which are below the naïve bound is given in the column ‘fraction1’ whereas the median values of the frequency estimates are in the following. The next two columns contain similar information, but for a different test set. This time all singletons of *population 1* are considered. The rationale is that singletons are likely candidates for unseen haplotypes; in a different sample of the same population these are likely candidates for not being sampled. To be specific, consider Table 4 and the case *n = 50*. The naïve bound is 1/51 = 0.0196. The ‘median1’ estimates are below this value and decreases as gradually more distant test sets are considered corresponding to Scenario 1–3. The singleton test leads to a lower fraction of haplotypes below the threshold *1/(n+1)* and to higher frequency estimates (‘median2’); the reason is that these samples are closer to the population for which they are considered unseen. For the singleton test set there should be no systematic differences depending on scenario and only small stochastic differences are observed. The estimates are relatively stable for both test data (unseen and singletons) and increasingly stable as larger data sets are being used. The precise figures are by necessity somewhat speculative and there will also be some small difference depending on parameter settings

**Table 4:** Summary of the results of the simulation part of Example 5.

	n	naïve bound	fraction1	median1	fraction2	median2
Scenario 1	50	0.0196	0.87	0.01698	0.52	0.01858
Scenario 2	50	0.0196	1.00	0.01187	0.52	0.01861
Scenario 3	50	0.0196	1.00	0.00906	0.54	0.01861
Scenario 1	100	0.0099	1.00	0.00718	0.52	0.00939
Scenario 2	100	0.0099	1.00	0.00524	0.52	0.00940
Scenario 3	100	0.0099	1.00	0.00418	0.52	0.00940
Scenario 1	400	0.0025	1.00	0.00155	0.52	0.00237
Scenario 2	400	0.0025	1.00	0.00119	0.52	0.00237
Scenario 3	400	0.0025	1.00	0.00099	0.52	0.00237

The naïve bound, the estimate *1/(n+1)*, provides for a classical alternative to the estimates given in columns ‘median1’(based on unseen haplotypes generated from a different population) and ‘median2’ (based on singletons). Further details are provided in text.

**Figure 1:**
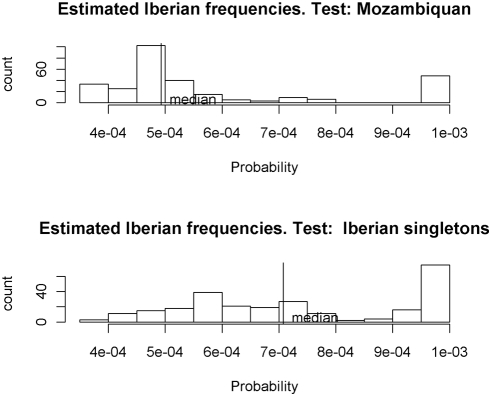
Estimates of frequencies of unseen Iberian haplotypes. The values were calculated following the PCA approach. The test set for the upper panel are those haplotypes of the Mozambique database which are unseen in the Iberian. The singletons in the Iberian database are used as the test set in the lower panel.

We next apply the methods to real data summarised in Table 3. For this purpose we used the Iberian database and the Mozambique haplotypes not seen in the Iberian database. We divided the Iberian database into two parts: *Ib.singles* (261 haplotypes occurring only once) and *Ib.rest* (the remaining 755 haplotypes). We performed PCA based on *Ib.rest* and the number of principal components was 18, following a requirement to explain 80% of the variance. The average distance based on the PCA-transformed *Ib.rest* haplotypes was *d̅* = 0.22, where we used the distance defined in (10). There were 286 Mozambiquan haplotypes not seen in the Iberian database. For 238 of the 286 haplotypes (83.2%) the distance exceeded the average and we estimated the probabilities from (11). Figure 1 shows the distribution of the probabilities. In the upper panel, distances are based on the samples from Mozambique while the lower panel is based on singletons from the Iberian database. As expected, the probabilities were lower for the samples from Mozambique.

### Alternative approaches including saturation curves

There are many different approaches that can be tried in addition to those we have presented. We will not discuss what we consider to be minor variants that may or may not work better for specific data, but rather discuss approaches differing more substantially. A proposal based on saturation curves was formulated in [37]. Similar approaches have been suggested in various applications, see [16] for a review of some of these. The saturation approach may be parametric as in [37] where *f(x) = ax/(b+x)* is used (this corresponds to the Michaelis-Menten function, but this name is of no relevance to our application and is not mentioned in [37]). The interpretation of the function is as follows: for a sample of size *x*, the number of different haplotypes is estimated as *f(x)*. The parameters *a* and *b* must be estimated from the observed saturation curve and we have used the *nls* (nonlinear least squares) function in R. The saturation depends on the order of the haplotypes as do the parameter estimates. A practical solution to this problem is to repeat estimation for a number of randomly permutated orderings and present average values.

#### Example 6

**Figure 2:**
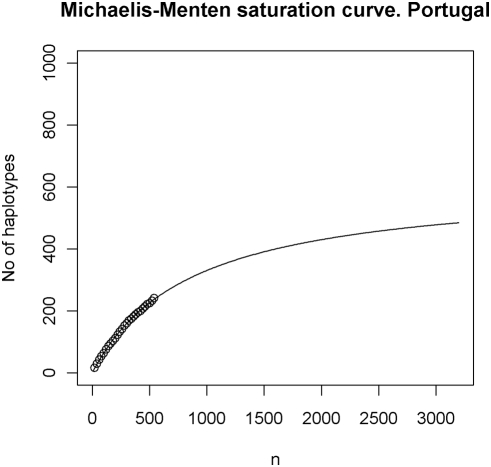
The saturation curve for the Portuguese database (see Table 3) based on the Michaelis-Menten function.

For this example, we will use the 540 haplotypes in the Portuguese database (Table 3). This allows comparison with [37], as the data differs only slightly. We permutated the order of the haplotypes 100 times and each time we estimated the *a* and *b* of *f(x) = ax/(b+x)*. The average estimate of *a* was 584.7 and this is a reasonable abundance estimate since it corresponds to the limit as *x* approaches infinity and because this value is also almost obtained for large, but finite values such as *x* = 100000. Our other estimates are 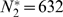 and 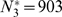. The estimate for *a* depends crucially on the order of the haplotypes, and values between 441 and 857 were obtained for the simulations. The resulting function with extrapolation up to 3200 is shown in Figure 2.

There is also an extensive general literature related to the problem of detecting a new species. Several recent papers extend the so-called Starr estimator introduced in [38]. Some more specific calculations related to haplotypes are provided by C. Brenner http://dna-view.com/haplofreq.htm.

## Discussion

We aimed to explore several statistical problems concerning databases of haplotype data (particularly, mtDNA and Y-chromosome) that can be useful for population, clinical, and forensic genetic applications. To our knowledge this is the first attempt to provide pragmatic solutions to these problems. We have facilitated software that implements these approaches and we also offer recommendations for particularly common situations in several genetic contexts. In particular, three estimators for haplotype abundance were proposed and tested. 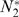 and 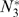 perform better than 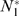 whereas the difference between 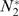 and 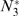 is smaller; the latter normally leads to higher estimates and appears to be better based on a simulation experiment reported in Tables 1 and 2.

One could use the approach proposed here to determine a lower bound on the fraction of unseen haplotypes in terms of the number of singletons *f_1_*. Sampling should then continue until 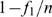 exceeds some prescribed lower level. Similar methods can also be used *a posteriori* to evaluate the coverage of a sample with respect to the reference population, and again, we may wish to set a threshold for the fraction of unseen haplotypes.

The saturation approach exemplified in Example 6 may work well in some cases, particularly if there is a justification for the parameterization of the saturation curve. However, these approaches have been criticized [9]. The methods may share the intrinsic problems of all extrapolation-based predictions: If extrapolation is carried beyond the validity of the model, ridiculous predictions may result. This may also be apparent from Figure 2: most of the data points lie on a relatively steep part of the curve and extrapolation of the curve depends crucially on the parameterization, which may be somewhat arbitrary. For instance, it is said that in some countries (e.g. Spain), women smoke progressively more cigarettes now than a decade ago in comparison to men for the same period of time; extrapolation would indicate that women will smoke considerably more than men in the near future; this conclusion sounds however very naïve.

Capture-recapture methods can be used to estimate coverage and abundance in much the same way as in classical studies designed to estimate the abundance of a specific species: A random sample of individuals is sampled and their mtDNA profiles are obtained. Next, a new sample is obtained from the population and based on the number of new haplotypes in this sample, estimates can be obtained. Although this approach is theoretically attractive, it is not practical in real life. There has been an explosion of methodological research in this area starting around 1985 according to [16].

A number of other useful applications based on coverage and abundance estimates can be listed and discussed. Here we only briefly add some comments on *haplotype diversity*. This and similar measures are frequently reported in the field of population genetics and molecular anthropology. However, these estimators require the number of haplotypes and the corresponding frequencies to be known, and as we have argued above, this is rarely the case. Rather we can represent the haplotype diversity as
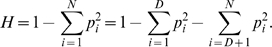



The last sum cannot be computed since it depends on the frequencies of the unseen haplotypes. However, based on our approach we can estimate *H*.

To sum-up, we have proposed different pragmatic approaches for estimating the coverage of databases and abundance of haplotypes and the frequency of unseen haplotypes. We have also discussed previous proposals. The methods discussed here have been tested on simulated and real data. We have also laid the foundations for other potential approaches based for instance on coalescence. The latter is however computationally demanding while the methods proposed here are easily implemented in conventional statistical packages such as R.

## Supporting Information

Table S1 Data set used for Example 1. There are four binary sites and 30 haplotypes. The first haplotype, haplo1, is a singleton, the second is the most prevalent occurring 12 times while there are four copies of the third (haplotypes 3,4, 16 and 18).
(0.04 MB DOC)

Table S2 The table is based on a large database of Y-chromosome data [2].There are 12727 haplotypes from 91 populations. Three different haplotypes are shown in followed by the most frequent haplotype occurring 661 times (5.2%). There are 2489 different haplotypes. The number of singletons is f1 = 1397 while the number of rare haplotypes, i.e., those occurring at most 10 times, is 4649. From these numbers the coverage is estimated as 1−1397/4649 = 0.7 with a 95% confidence interval ranging from 0.68 to 0.72.
(0.03 MB DOC)

## References

[1] Salas A, Richards M, De la Fé T, Lareu MV, Sobrino B (2002). The making of the African mtDNA landscape.. Am J Hum Genet.

[2] Roewer L, Krawczak M, Willuweit S, Nagy M, Alves C (2001). Online reference database of European Y-chromosomal short tandem repeat (STR) haplotypes.. Forensic Sci Int.

[3] Carracedo A, Bär W, Lincoln P, Mayr W, Morling N (2000). DNA commission of the international society for forensic genetics: guidelines for mitochondrial DNA typing.. Forensic Sci Int.

[4] Gill P, Brenner C, Brinkmann B, Budowle B, Carracedo A (2001). DNA Commission of the International Society of Forensic Genetics: recommendations on forensic analysis using Y-chromosome STRs.. Forensic Sci Int.

[5] Crawford DC, Nickerson DA (2005). Definition and clinical importance of haplotypes.. Annu Rev Med.

[6] Bandelt H-J, Kong QP, Parson W, Salas A (2005). More evidence for non-maternal inheritance of mitochondrial DNA?. J Med Genet.

[7] Marchini J, Cutler D, Patterson N, Stephens M, Eskin E (2006). A comparison of phasing algorithms for trios and unrelated individuals.. Am J Hum Genet.

[8] Fisher R, Corbet A, Williams C (1943). The relation between the number of species and the number of individuals in a random sample of animal population.. Journal of Animal Ecology.

[9] Bunge K, Fitzpatrick M (1993). Estimating the number of species: A review.. JASA.

[10] Haas P, Stokes L (1998). Estimating the number of classes in a finite population.. JASA.

[11] Efron B, Thisted R (1976). Estimating the number of species: How many words did Shakespeare know.. Biometrika.

[12] Robbins H (1956). An empirical bayes approach to statistics.

[13] Huang SP, Weir BS (2001). Estimating the total number of alleles using a sample coverage method.. Genetics.

[14] Chao A, Lee S-W (1992). Estimating the number of classes via sample coverage.. Journal of the American Statistical Association.

[15] Good I (1953). On the population frequencies of species and the estimation of population parameters.. Biometrika.

[16] Chao A (2005). Species richness estimation.

[17] Esty W (1983). A normal limit law for a nonparametric estimator of the coverage of a random sample.. Annals of Statistics.

[18] Haas P, Stokes L (1998). Estimating the number of classes in a finite population.. JASA.

[19] Nordborg M (2001). Coalescent theory.

[20] Pfeiffer H, Brinkmann B, Huhne J, Rolf B, Morris AA (1999). Expanding the forensic German mitochondrial DNA control region database: genetic diversity as a function of sample size and microgeography.. Int J Legal Med.

[21] Pfeiffer H, Forster P, Ortmann C, Brinkmann B (2001). The results of an mtDNA study of 1200 inhabitants of a German village in comparison to other Caucasian databases and its relevance for forensic casework.. International Journal of Legal Medicine.

[22] Helgason A, Sigurðardóttir S, Gulcher JR, Ward R, Stefánsson K (2000). mtDNA and the origin of the Icelanders: deciphering signals of recent population history.. Am J Hum Genet.

[23] Pereira L, Prata MJ, Amorim A (2000). Diversity of mtDNA lineages in Portugal: not a genetic edge of European variation.. Ann Hum Genet.

[24] González AM, Brehm A, Pérez JA, Maca-Meyer N, Flores C (2003). Mitochondrial DNA affinities at the Atlantic fringe of Europe.. Am J Phys Anthropol.

[25] Bertranpetit J, Sala J, Calafell F, Underhill PA, Moral P (1995). Human mitochondrial DNA variation and the origin of Basques.. Annals of Human Genetics.

[26] Richards M, Côrte-Real H, Forster P, Macaulay V, Wilkinson-Herbots H (1996). Paleolithic and neolithic lineages in the European mitochondrial gene pool.. American Journal of Human Genetics.

[27] Côrte-Real HBSM, Macaulay VA, Richards MB, Hariti G, Issad MS (1996). Genetic diversity in the Iberian peninsula determined from mitochondrial sequence analysis.. Ann Hum Genet.

[28] Crespillo M, Luque JA, Paredes M, Fernández R, Ramirez E (2000). Mitochondrial DNA sequences for 118 individuals from northeastern Spain.. Int J Legal Med.

[29] Salas A, Comas D, Lareu MV, Bertranpetit J, Carracedo Á (1998). mtDNA analysis of the Galician population: a genetic edge of European variation.. Eur J Hum Genet.

[30] Mao CX (2004). Predicting the conditional probability of discovering a new class.. JASA.

[31] Plaza S, Salas A, Calafell F, Corte-Real F, Bertranpetit J (2004). Insights into the western Bantu dispersal: mtDNA lineage analysis in Angola.. Hum Genet.

[32] Balding DJ (2005). Weight-of-Evidence for Forensic DNA Profiles:.

[33] Curran JM, Buckleton JS, Triggs CM, Weir BS (2002). Assessing uncertainty in DNA evidence caused by sampling effects.. Sci Justice.

[34] Wilson I, Weale M, Balding D (2003). Inferences from DNA data: population histories, evolutionary processes and forensic match probabilities.. JR Statist Soc A.

[35] Krawczak M (2001). Forensic evaluation of Y-STR haplotype matches: a comment.. Forensic Sci Int.

[36] Egeland T, Bovelstad HM, Storvik GO, Salas A (2004). Inferring the most likely geographical origin of mtDNA sequence profiles.. Ann Hum Genet.

[37] Pereira L, Cunha C, Amorim A (2004). Predicting sampling saturation of mtDNA haplotypes: an application to an enlarged Portuguese database.. Int J Legal Med.

[38] Starr N (1979). Linear estimation of the probability of discovering a new species.. The Annals of Statistics.

