# Supplementary material for: Estimating Haplotype Frequency and Coverage of Databases
Source: PLoS One. 2008 Dec 22;3(12):e3988. doi: 10.1371/journal.pone.0003988 (PMC2602601; doi:10.1371/journal.pone.0003988)
Supplement: Table S2 — The table is based on a large database of Y-chromosome data [2].There are 12727 haplotypes from 91 populations. Three different haplotypes are shown in followed by the most frequent haplotype occurring 661 times (5.2%). There are 2489 different haplotypes. The number of singletons is f1 = 1397 while the number of rare haplotypes, i.e., those occurring at most 10 times, is 4649. From these numbers the coverage is estimated as 1−1397/4649 = 0.7 with a 95% confidence interval ranging from 0.68 to 0.72. (0.03 MB DOC) [file pone.0003988.s002.doc]

| **DYS19** | **DYS389i** | **DYS389ii** | **DYS390** | **DYS391** | **DYS392** | **DYS393** | **pop.name** |
| --- | --- | --- | --- | --- | --- | --- | --- |
| 12 | 13 | 30 | 24 | 10 | 11 | 13 | Albania |
| 14 | 13 | 29 | 24 | 11 | 13 | 13 | Madrid,Central-East Spain |
| 17 | 13 | 30 | 25 | 10 | 11 | 13 | Anatolia,Turkey |
| 14 | 12 | 29 | 23 | 10 | 11 | 14 | Most frequent haplotype |
