# Supplementary material for: Estimating Haplotype Frequency and Coverage of Databases
Source: PLoS One. 2008 Dec 22;3(12):e3988. doi: 10.1371/journal.pone.0003988 (PMC2602601; doi:10.1371/journal.pone.0003988)
Supplement: Supplementary file 2 [file pone.0003988.s001.doc]

haplotype	site1	site2	site3	site4
haplo1	1	0	0	1
haplo2	0	0	0	0
haplo3	1	1	0	0
haplo4	1	1	0	0
haplo5	1	0	1	1
haplo6	1	1	0	1
haplo7	1	0	0	0
haplo8	1	0	0	0
haplo9	1	0	0	0
haplo10	0	1	0	0
haplo11	0	1	0	0
haplo12	0	1	0	0
haplo13	0	1	0	1
haplo14	0	1	0	0
haplo15	1	0	0	0
haplo16	1	1	0	0
haplo17	0	1	0	0
haplo18	1	1	0	0
haplo19	0	0	0	0
haplo20	0	0	0	0
haplo21	0	0	0	0
haplo22	1	1	1	1
haplo23	0	0	0	0
haplo24	0	0	0	0
haplo25	0	0	0	0
haplo26	0	0	0	0
haplo27	0	0	0	0
haplo28	0	0	0	0
haplo29	0	0	0	0
haplo30	0	0	0	0
